# The impact of living donor hypertension on later function of kidney graft in kidney transplantation

**DOI:** 10.3389/fmed.2025.1686473

**Published:** 2025-12-03

**Authors:** Sophie Huegli, David Jaques, Noemie Grouzmann, Theodore Pasquier, Patricia Hirt-Minkowski, Solange Moll, Sophie de Seigneux, Belen Ponte, Fadi Haidar

**Affiliations:** 1 Division of Nephrology and Hypertension, Department of Medicine, Valais Romand Hospital (CHVR), Sion, Switzerland; 2 Division of Nephrology and Hypertension, Department of Medicine, Geneva University Hospitals, Geneva, Switzerland; 3 Division of Internal Medicine, Department of Medicine, Geneva University Hospitals, Geneva, Switzerland; 4 Living Kidney Donor Registry (SOL-DHR), University Hospital Basel, Basel, Switzerland; 5 Division of Pathology, Geneva University Hospitals, Geneva, Switzerland

**Keywords:** living donor, kidney transplant, hypertension, eGFR, fibrosis

## Abstract

**Introduction:**

The impact of hypertension (HT) of living donors on later kidney graft function remains insufficiently understood.

**Methods:**

We retrospectively reviewed recipients of living donor (LD) kidney transplant (KTX) at a single tertiary center from January 2003 to December 2021 with a follow-up until December 2022. LD blood pressure (BP) values obtained with office measurement and 24 h ambulatory BP monitoring (ABPM) were the main predictors, while recipient estimated glomerular filtration rate (eGFR) and proportion of kidney fibrosis on biopsies were the outcomes. Multivariate analyses were adjusted for pre-transplant donor characteristics: age, sex, ethnicity, body mass index (BMI) and eGFR.

**Results:**

212 LD KTX recipients were included, with mean age 51 years. 133 were women (62.7%). 73 (34.4%) LD were hypertensive based on office BP. In a sub-group of 112 LD with ABPM, 64 (57.1%) were hypertensive. Office systolic blood pressure (SBP) was negatively associated with eGFR at 6 months, 1 year, 5 years and 10 years (*p* < 0.05). Office SBP was positively associated with kidney fibrosis at 1 year (*p* < 0.05). Those associations were not significant after multivariate adjustment.

**Discussion:**

In conclusion, while an adverse impact of LD HT on later kidney function and fibrosis was measured, this effect seemed negligible after accounting for other more relevant clinical characteristics.

## Introduction

Kidneys are the most commonly transplanted organ in the world. In Switzerland, 250–300 kidney transplants (KTX) are performed each year, with 35–40% of them coming from living donors (LD) (1, 2). KTX from LD is known to have a better prognosis than KTX from deceased donors (3). In the current context of global organ scarcity, the criteria for deceased donors have been expanded, including the acceptance of “marginal” donors, such as elderly or hypertensive patients.

The main factors influencing graft renal function outcomes are donor age and donor estimated glomerular filtration rate (eGFR). Therefore, kidney function is carefully assessed in LD prior to donation. An eGFR <60 mL/min is a contraindication for living kidney donation, as is an albuminuria level exceeding >30 mg/day based on a single urine collection (4).

LD selection is a crucial step in KTX. The mean age of LD is 53 years in Switzerland and is constantly increasing. Hypertensive LD are commonly accepted for donation in Europe, provided they have well controlled blood pressure (BP) with a maximum of 2 antihypertensive drugs, and there is no evidence of target organ damage (5). It is important to note that renal damage is only assessed by eGFR and albuminuria; no pre-donation kidney biopsy is required to detect subclinical nephroangiosclerosis. Hypertensive LD represent approximately 15% of LD in Switzerland. As in the general population in Europe, hypertension (HT) in LD is defined as office BP ≥ 140/90 mmHg, home BP ≥ 135/85 mmHg or mean BP on ambulatory blood pressure monitoring (ABPM) ≥ 130/80 mmHg (6). Past studies on hypertensive LD have used these definitions interchangeably. At our tertiary center in Geneva, all LD candidates undergo pre-donation ABPM to screen for HT or to evaluate BP control under antihypertensive treatment. Surprisingly, however, international guidelines do not recommend ABPM for LD selection (4), while HT societies’ guidelines recommend ABPM as a first-line method for diagnosing or confirming HT in the general population (6).

HT causes early renal structural changes in kidney biopsies, known as nephroangiosclerosis, which include arteriolar hyalinosis, glomerular sclerosis, tubular atrophy, and interstitial fibrosis. Reperfusion biopsies (performed at the time of KTX) from hypertensive donors show more signs of nephroangiosclerosis than biopsies from normotensive donors (7). In the general population, nephroangioclerosis is associated with an increased risk of chronic kidney disease (CKD), albuminuria, and, in severe cases, end-stage kidney disease (ESKD). A Mayo Clinic cohort of over 2000 LD and KTX recipients demonstrated that signs of nephroangiosclerosis on reperfusion biopsies, particularly the percentage of interstitial fibrosis, were associated with faster eGFR decline, and a moderate increase in the risk of graft loss (3). A meta-analysis including studies on kidney grafts from both LD and deceased donors showed that pre-existing donor HT modestly increased the risk of CKD and graft loss (8). However, there was significant heterogeneity between the studies, likely due to publication bias. There was no effect on recipient mortality (8). In contrast, a retrospective study of the American national registry, which included more than 40′000 patients, found no association between the development of early post-donation HT in LD and recipient eGFR decline (9). Therefore, the existing literature is not conclusive regarding the long-term prognosis of kidney grafts from hypertensive LD. Furthermore, to our knowledge, all existing studies have focused solely on reperfusion biopsies, and no studies have been conducted with biopsies taken 1 year post-KTX.

In our study, we aimed to explore the renal prognosis of recipients of kidney transplants from hypertensive and normotensive LD, by examining the association between BP and eGFR at 6 months, 1 year, 5 years, and 10 years post-KTX, as well as interstitial fibrosis on kidney biopsies at time of KTX and 1 year later.

## Materials and methods

### Population

We conducted a retrospective, monocentric study of all adults KTX LD and their recipients at a tertiary center, the Geneva University Hospitals, in Switzerland, between January 1st 2003 and December 31st 2021, with follow-up until December 31st 2022. The minimum follow-up for inclusion was 1 year. Exclusion criteria included crossover kidney donation involving at least one LD or recipient from another center, for data availability reasons, as well as recipient age <18 years.

We report no case of diabetic LD, LD with eGFR <60 mL/min or LD with albuminuria >30 mg/day, as these are exclusion criteria for kidney donation.

All LD provide informed consent for the use of their data for research purposes when they are included in the Swiss Organ Living-Donor Health Registry (SOL-DHR) at the time of donation. KTX recipients also give informed consent for the use of their data when included in the Swiss Transplant Cohort Study (STCS), which has been including patients since 2007. For the KTX recipients transplanted between 2003 and 2007 who are not included in STCS (*n* = 41), our Ethics Committee granted an exception to the requirement for individual informed consent, as this was deemed disproportionate. Given that our study is retrospective and poses no additional risk to the included patients, and that some patients were already deceased at the beginning of the study, this exception was justified.

Our study was conducted in accordance with the Declaration of Helsinki and was approved by our local Ethics Committee (CCER n° 2022-01572).

### Collected variables

We retrospectively collected demographic, clinical and laboratory data concerning LD pre-donation, as well as demographic, clinical, laboratory and kidney biopsy data for KTX recipients at 6 months, 1 year, 5 years and 10 years post-KTX. A sub-group of LD underwent ABPM pre-donation, following a change of practice in our department. LD data was extracted from the SOL-DHR and electronic patient records, while recipient data was obtained from electronic patient records.

Office BP measurements were taken with the patient seated for at least 5 min in a quiet room, using a validated upper-arm blood pressure monitor. Only one BP per patient was recorded, in accordance with current practice. Office HT was defined as office BP > 140/90 mmHg and/or at least one antihypertensive drug. ABPM measurements were obtained using a validated DiaSys monitor, with BP recorded every 20 min during the day and every 30 min during the night. HT on ABPM was defined as a mean BP over 24 h > 130/80 mmHg, daytime BP > 135/85 mmHg, or nighttime BP > 120/70 mmHg, and/or at least one antihypertensive drug. All ABPM in our study had sufficient measurements to be interpretable (at least 14 daytime and 7 nighttime measurements). The nocturnal dip was considered as present whenever its value was ⩾ 10%. A single nephrologist (BP) interpreted all ABPM results.

All laboratory values were performed centrally at the local laboratory. We used the CKD-EPI 2021 equation for eGFR estimation. For albuminuria, we relied on a single urine collection estimate, considering albuminuria to be significant if >30 mg/day.

The same pathologist (SM) examined all renal biopsies and specified the percentage of interstitial fibrosis. Interstitial fibrosis was assessed semi-quantitatively using trichrome staining.

### Statistical analysis

Continuous variables are presented as mean with standard deviation (SD). Categorical variables are presented as numbers and relative frequencies. Differences between groups were assessed using student T- test and chi-square test for continuous and categorical variables, respectively. Transversal and longitudinal analyses were conducted using uni- and multivariate linear regression models using SBP and DBP as the main independent variables and eGFR as the dependent variable. Models were adjusted for donors’ characteristics known to affect recipients’ kidney function: LD age, sex, ethnicity, BMI and pre-donation eGFR. Mixed models were used to account for time-repetition when necessary. The group variable was represented by patient identification, while a random effect was applied to the intercept. A potential interaction effect between blood pressure and the time variable was also considered. Instead of continuous SBP or DBP we also performed the analyses using HT (yes/no) as the variable of interest.

All analyses were carried out using Stata 17 software (StataCorp, 4,905 Lakeway Drive, College Station, Texas 77,845 USA). Statistical significance was set at a *p*-value of 0.05 in all analyses. No imputation on missing data was made.

## Results

We included 212 donor/recipient pairs (424 patients). Among the 212 LD, 133 were women (62.7%) and 79 were men (37.3%), with a mean age of 51.6 (± 11.4) years. The mean pre-donation eGFR was 94.8 (± 14.3) mL/min/1.73m^2^, and the median pre-donation albuminuria was zero. Twenty-nine LD (13.7%) were taking at least one antihypertensive drug before donation. LD characteristics are shown in Table 1, stratified by subgroup: one subgroup of 110 LD had office BP (OBPM) measured only, and the other subgroup of 112 LD had office BP and ABPM measured. Compared to those with office BP only, those with office BP and ABPM were more frequently caucasian, had a slightly lower eGFR, had higher office systolic and diastolic BP and were much more likely to be taking an anti-hypertensive medication. Baseline fibrosis was 5% (0–10).

**Table 1:** Baseline pre-donation LD characteristics, according to BP measure available (*N* = 212).

	OBPM only (*N* = 100) (47.1%)	OBPM + ABPM (*N* = 112) (52.8%)	*p* value
Age	50.7 +/− 12.3	52.5 +/− 10.6	0.269
Gender (men)	37 (37.0%)	42 (37.5%)	0.940
Ethnicity (caucasian)	72 (72.0%)	74 (66.0%)	**0.002**
BMI (kg/m2)	24.8 +/− 3.4	25.5% +/− 3.4	0.141
eGFR (mL/min/1,73 m2)	97.6 +/− 13.3	92.5 +/− 14.9	**0.010**
Albuminuria >30 mg/24 h	1 (1.0%)	2 (1.8%)	0.633
Antihypertensive treatment	3 (3.0%)	26 (23.2%)	**<0.001**
Office SBP	125.8 +/− 16.0	136.6 +/− 18.9	**<0.001**
Office DBP	74.0 +/− 10.7	78.8 +/− 11.0	**0.001**
Daytime SBP (ABPM)	–	122.5 +/− 12.8	–
Daytime DBP (ABPM)	–	79.7 +/− 7.8	–
Nighttime SBP (ABPM)	–	107.8 +/− 12.4	–
Nighttime DBP (ABPM)	–	69.1 +/− 8.1	–
No systolic dipping (ABPM)	–	44 (39.2%)	–
No diastolic dipping (ABPM)	–	37 (33.0%)	–

LD: living donors, BMI: body mass index, eGFR: estimated glomerular filtration rate (CKD-EPI), SBP: systolic blood pressure, DBP: diastolic blood pressure, ABPM: ambulatory blood pressure monitoring, OBPM: office blood pressure monitoring.

*p* values in bold type indicate *p* value <0.05.

Office HT was found in 73 (34.4%) of 212 LD, with no significant difference between men and women. A subgroup of 112 LD underwent pre-donation ABPM: among them 64 (57.1%) were hypertensive according to ABPM (>130/80 mmHg) and 51 (45.5%) were hypertensive according to office BP (>140/90 mmHg). There were 33 (29.5%) normotensive patients, 28 (25%) patients with masked HT, 15 (13.4%) patients with white-coat HT and 36 (32.1%) patients with sustained HT (HT according to both office and ABPM BP), as shown in Supplementary Data Sheet 3. Moreover, systolic and diastolic dipping was preserved in 68 (60.7%) and 75 (66.9%) patients, respectively. Office BP and ABPM measurements were well correlated in the subgroup of patients who had both (Supplementary Data Sheets 1, 2).

### Association between blood pressure and eGFR

**Figure 1:**
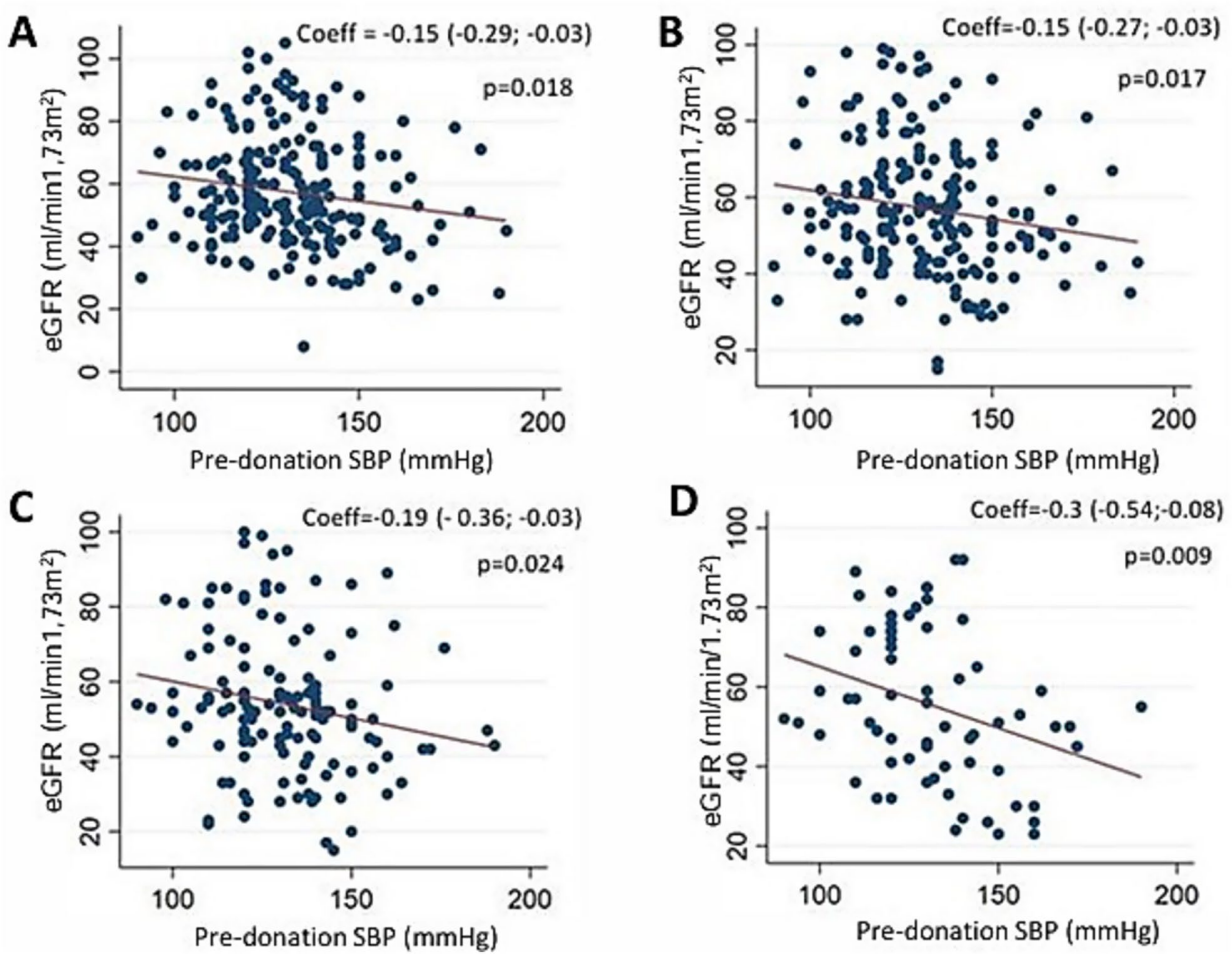
Association between LD SBP and pre-donation recipient eGFR (CKD-DPI) in univariate linear regression. **(A)** 6 months post-KTX, *N* = 206, *R*^2^ = 0.02. **(B)** 1 year post-KTX, *N* = 202, *R*^2^ = 0.02. **(C)** 5 years post-KTX, *N* = 136, *R*^2^ = 0.03. **(D)** 10 years post-KTX, *N* = 67, *R*^2^ = 0.1 LD: living donors, SBP: systolic blood pressure, KTX: kidney transplantation.

In univariate linear regression analyses, we found inverse associations between pre-donation LD SBP and recipient eGFR at 6 months (coefficient −0.15 [95% CI: −0.29; −0.03] *p* = 0.018), 1 year (−0.15 [95% CI: −0.27; −0.03], *p* = 0.017, *N* = 202), 5 years (−0.19 [95% CI: −0.36; −0.03], *p* = 0.024, *N* = 136), and 10 years (−0.30 [95% CI: −0.53; −0.08], *p* = 0.009, *N* = 67) (Figure 1).

However, there was no association between LD pre-donation DBP and recipient eGFR at any follow-up time.

Table 2 presents the result from the adjusted analyses for both SBP and DBP at 6 months. After adjustment, the association between SBP and eGFR at 6 months was no longer significant. However, eGFR was inversely associated with LD age (−0.37; 95% CI: −0.6; −0.14) and positively associated with BMI (1.0; 95% CI: 0.37; 1.62) and pre-donation eGFR (0.29; 95% CI: 0.12; 0.47). LD sex and ethnicity were not associated with eGFR at 6 months.

**Table 2:** Association between pre-donation LD SBP and DBP, and recipient eGFR at 6 months post-KTX in multivariate linear regression.

	Systolic BP			Diastolic BP		
	Coefficient	Confidence interval (95%)	*p* value	Coefficient	Confidence interval (95%)	*p* value
SBP	−0.06	(−0.18; 0.05)	0.277	–		
DBP	–			0.05	(−0.15;0.25)	0.64
Age	−0.37	(−0.6; −0.14)	**0.001**	−0.39	(−0.62; −0.17)	**0.001**
Sex	4.1	(−0.28; 8.6)	0.066	3.86	(−0.64; 8.4)	0.09
Ethnicity	−0.38	(−1.02; 0.26)	0.239	−0.36	(−0.99; 0.27)	0.26
BMI	1.0	(0.37; 1.62)	**0.002**	0.92	(0.27; 1.57)	**0.006**
eGFR	0.29	(0.12; 0.47)	**0.001**	0.31	(0.14; 0.49)	**<0.001**

Adjusted for LD age, sex, ethnicity, BMI and eGFR. *N* = 202. Sex: female = 0/male = 1, Ethnicity: caucasian = 0/black = 1.

LD: living donors, BP: blood pressure, BMI: body mass index, eGFR: estimated glomerular filtration rate (CKD-EPI), SBP: systolic blood pressure, KTX: kidney transplantation.

*p* values in bold type indicate *p* value <0.05.

Multivariate analyses at 1, 5 and 10 years were similar to those presented for eGFR at 6 months (data not shown): there was no association between either SBP or DBP and eGFR after adjustment. Similar associations were observed between LD age, BMI, pre-donation eGFR and recipient eGFR.

**Figure 2:**
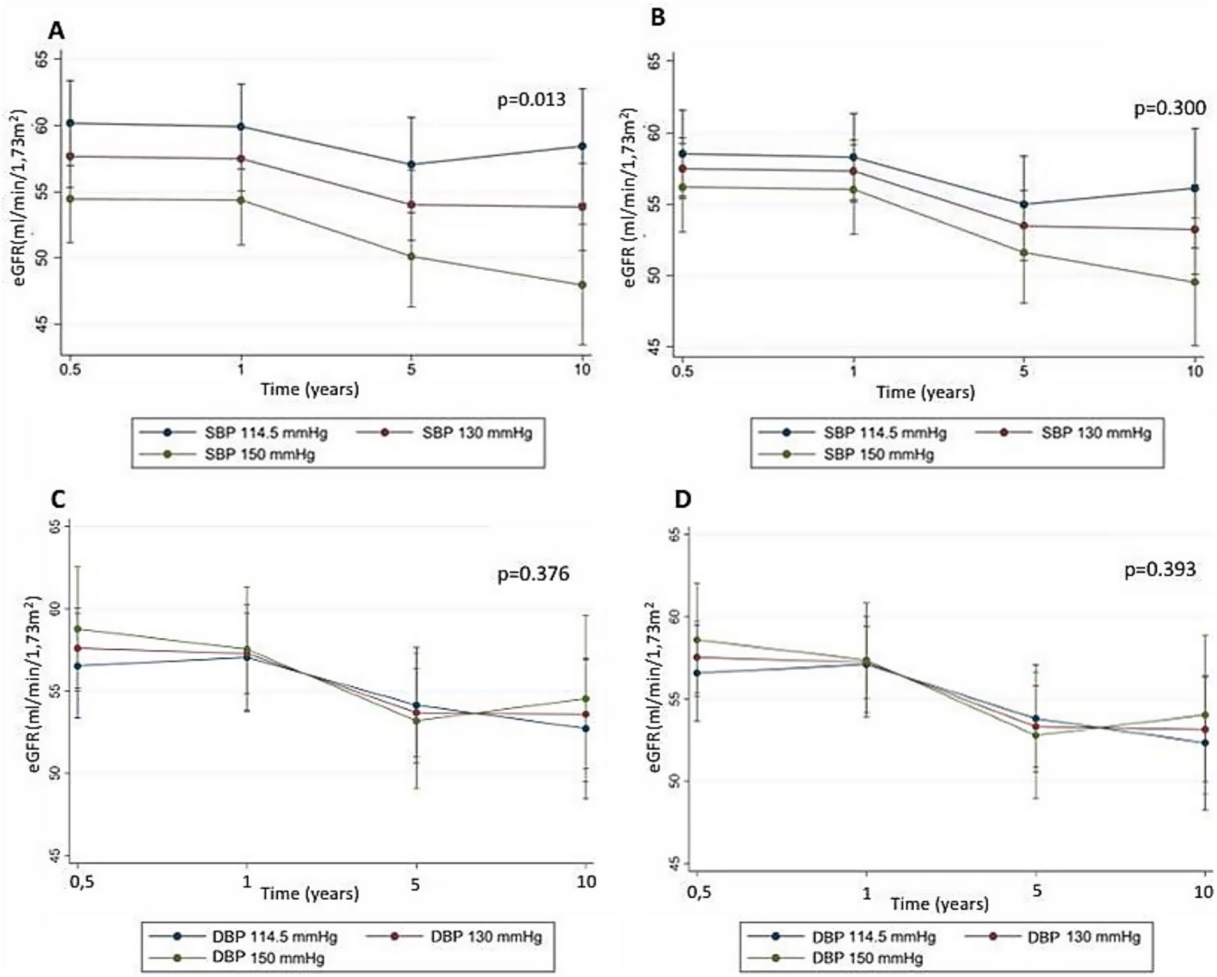
Mixed linear univariate regression of recipient eGFR (CKD-EPI) variation over time according to LD pre-donation BP, at 6 months, 1 year, 5 years and 10 years post-KTX. **(A)** Univariate model for SBP, *N* = 209. **(B)** Multivariate model for SBP, adjusted for pre-donation LD age, sex, ethnicity, BMI, eGFR, *N* = 205. **(C)** Univariate model for DBP, *N* = 209. **(D)** Multivariate model for DBP, adjusted for pre-donation LD age, sex, ethnicity, BMI, eGFR, *N* = 205.

Figure 2 shows a longitudinal model of variation in recipient eGFR, taking into account all time points (6 months, 1 year, 5 years and 10 years), according to LD SBP and DPB tertiles, using mixed linear regression. The univariate analysis for SBP (part A) shows a negative association between LD SBP and recipient eGFR, with a coefficient of −0.16 (95% CI: −0.29; −0.03, *p* = 0.013, *N* = 209). The multivariate analysis for SBP (B) no longer shows this association (−0.07 (95% CI: −0.18; 0.05), *p* = 0.283, *N* = 205). The univariate analysis for DPB (C) finds no association between DPB and eGFR (*p* = 0.376), and the multivariate analysis for DPB (D) also finds no association (*p* = 0.393).

We also repeated the analyses using the presence of LD HT as a categorical variable. In the univariate analysis, the presence of HT was associated with a lower eGFR of −5.8 mL/min (95% CI: −10.8; −0.93) at 6 months. However, after adjustment there was no longer any association. Analyses using a different definition of HT, based only on office BP, yielded similar results.

In the subgroup of 112 LD with ABPM results, there was also no association between the presence of HT and recipient eGFR at 6 months, likely due to lack of statistical power (coefficient −4.1 mL/min (95% CI: −11.1; 2.94)).

### Association between blood pressure and fibrosis percentage

Mean fibrosis on reperfusion biopsies was 5 (0–10) %, and 15 (10–20) % 1 year post-KTX.

**Figure 3:**
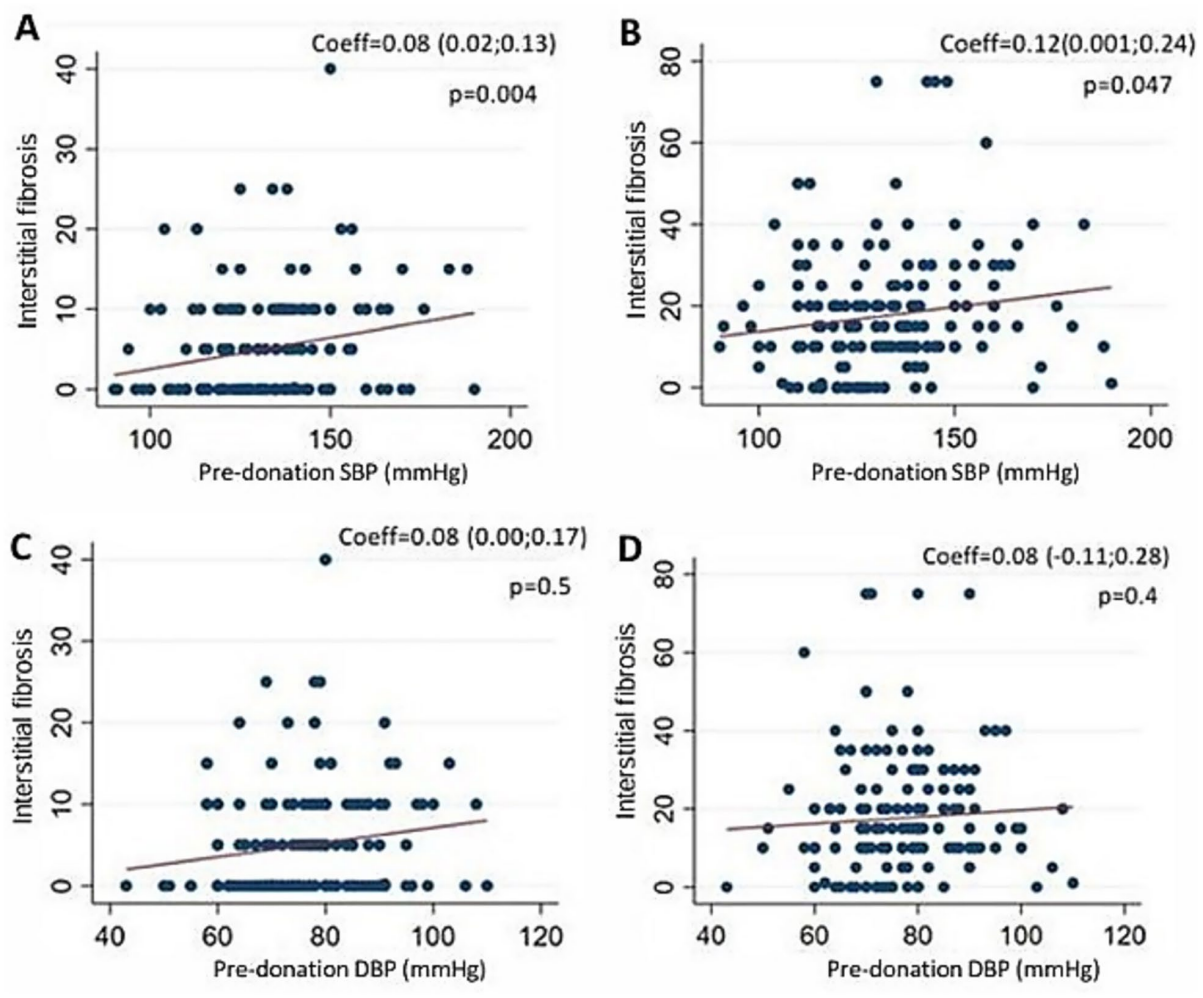
Association between LD SBP and DBP and percentage of graft interstitial fibrosis at TO and 1 year post-KTX in univariate linear regression. **(A)** Association between SBP and interstitial fibrosis at TO, *N* = 166, *R*^2^ = 0.05. **(B)** Association between SBP and interstitial fibrosis 1 year post-KTX, *N* = 173, *R*^2^ = 0.02. **(C)** Association between DBP and interstitial fibrosis at TO, *N* = 166, *R*^2^ = 0.02. **(D)** Association between DBP and interstitial fibrosis 1 year post-KTX, *N* = 173, *R*^2^ = 0.00 LD: living donors, SBP: systolic blood pressure, DPB: diastolic blood pressure, KTX: kidney transplantation, T0: time of kidney transplant.

In univariate regression analysis, there was a positive association between pre-donation LD SBP and the percentage of kidney graft interstitial fibrosis on biopsies at the time of KTX (*N* = 166) and 1 year later (*N* = 173). The coefficients were 0.08 (95% CI: 0.02; 0.13; *p* = 0.004), and 0.2 (95% CI: 0.001; 0.24: *p* = 0.047), respectively. However, there was no association between DBP and fibrosis at time of KTX and 1 year later: coefficients 0.08 (95% CI: 0.00; 0.17; *p* = 0.50) and 0.08 (95% CI: −0.11; 0.28; *p* = 0.401), respectively. Graphs are shown in Figure 3.

Multivariate analyses at time of KTX and at 1 year also showed no association between SBP and fibrosis. At KTX, LD age, ethnicity and pre-donation BMI were not associated with fibrosis; however male sex was associated with an increased interstitial fibrosis percentage, with a coefficient of 2.86 (95% CI: 0.8; 4.92; *p* = 0.007). Conversely, pre-donation LD eGFR was associated with a decrease in fibrosis: -0.11 (95% CI: −0.19; −0.26; *p* = 0.011). At 1 year post-KTX, LD sex and eGFR were no longer associated with fibrosis. In contrast, LD age and African ethnicity were both associated with increased fibrosis.

## Discussion

In our study, we aimed to determine whether HT in LD impacts recipient renal function, and kidney fibrosis in graft biopsies. The goal was to ascertain whether it is appropriate to continue accepting grafts from strictly selected hypertensive LD with well-controlled BP on a maximum 2 antihypertensive drugs, and without end-organ damage. This question is crucial in the global and Swiss context of organ shortage, especially since grafts from LD have a much better long-term prognosis than those from deceased donors (3). Our LD population was two-thirds female, with an average age of 51, which is comparable to the existing literature on LD in Western countries.

In our study, there was no association between office DBP and recipient eGFR. On the contrary, we found an association, in univariate analyses, between LD office SBP and recipient eGFR at 6 months, 1 year, 5 years and 10 years post-KTX. The presence of HT was associated with a 5.8 mL/min/1.73m^2^ lower eGFR at 6 months. This association accounted for approximately 2–3% of eGFR variability at 6 months, 1 year and 5 years, but 10% of eGFR variability at 10 years. However, LD SBP was no longer associated with recipient renal function, once potential confounding factors (LD age, BMI, sex, ethnicity and pre-donation eGFR) were accounted for in multivariate analyses. Altogether, these factors explained around 26% of eGFR variability at 6 months. Our findings are consistent with a large American study, which showed that kidney grafts from LD who developped HT within 2 years post-donation had the same risk of graft failure as those who did not (9). In contrast, a meta-analysis including LD and cadaveric donors showed a slight risk increase in graft failure for grafts from hypertensive donors, but no increase in mortality risk (8). There was significant heterogeneity between the studies, and studies as early as 1960 were included, which could explain the discrepancy with our results.

The most significant factor influencing variability in graft eGFR was LD age at time of donation, which has already been shown in other studies (3, 10). We also found an association between LD BMI and recipient eGFR, likely explained by greater nephron mass, as well as between LD eGFR and recipient eGFR. There was no association between LD sex or ethnicity and eGFR. Previous studies have shown that KTX from Black patients have a poorer long-term prognosis (3, 11), in part due to APOL1 genetic variants. Our study may suffer from a lack of power, as the number of Black patients (4.2% of LD) was too low to detect a significant difference in eGFR.

The subgroup of 112 LD who underwent ABPM included 13.4% patients with white-coat HT and 25% with masked HT, both of which are higher than in the general population. Indeed, a Swiss cohort reported a prevalence of 3% for white-coat HT and 16% for masked HT (12). A Mayo Clinic study conducted between 2012 and 2017 also reported a prevalence of 5.8% for white coat HT and 14.4% for masked HT (13). However, this was a younger population than the one described in our study, and it is well known that prevalence of both white coat and masked HT increases with age. Unfortunately, in the ABPM subgroup, we could not demonstrate an association between LD SBP or DBP and recipient eGFR, regardless of the time-period. This is likely due to insufficient statistical power, given the smaller number of patients in this subgroup. Very few previously published studies have included ABPM, leaving us with limited reliable data on the impact of the 24 h BP profile, or on the absence of dipping, on the renal function of recipients. In addition, the definition of HT in these studies has been heterogenous, often recording only SBP. New studies reporting standardized measurements of SBP and DBP, as well as studies including ABPM, are needed.

There was no association between DBP and fibrosis. However, we found an association between SBP and interstitial fibrosis on kidney biopsies at time of KTX and 1 year after in univariate analysis. At time of KTX, a 10 mmHg increase in SBP corresponded to a 0.07% increase in fibrosis. 1 year after KTX, a 10 mmHg increase in SBP corresponded to a 1.2% increase in fibrosis. SBP explained around 5% of the variability in interstitial fibrosis at time of KTX and 2% 1 year after. Previous studies have indicated that interstitial fibrosis at time of KTX is associated with a more rapid decline in renal function, as well as with a higher risk of graft loss (3, 14). In multivariate analyses, LD SBP was no longer associated with fibrosis after accounting for LD age, sex, ethnicity, BMI and eGFR. Together, these factors explained approximately 13% of the variability of interstitial fibrosis at time of KTX and 9.5% 1 year after, highlighting the challenge of predicting interstitial fibrosis given the multiple factors involved. The predictive factors found in multivariate analysis for fibrosis at time of KTX were male sex and pre-donation eGFR. LD age was not associated with fibrosis at time of KTX. 1 year after KTX, the predictive factors found in multivariate analysis were LD age and Black ethnicity.

There is limited data in the literature concerning the association between LD age and interstitial fibrosis. A small Japanese study involving 45 patients found no difference between LD over and under 60 years old regarding fibrosis on kidney biopsies at time of KTX and 1 and 2 years after (15). However, these results may not be generalizable to our predominantly Caucasian population.

The main strength of our study is the inclusion of all LD/recipient pairs which underwent KTX in Geneva between January 2003 and December 2021, including over 200 patients. Furthermore, a high proportion of LD were hypertensive (47–57%, depending on the definition of HT). Ours is the first study to investigate the relationship between LD HT and graft renal function in Switzerland. All LD had office BP measurements, and just over half had ABPM results. To our knowledge, no large-scale study on the subject has yet been published based on ABPM results.

We also collected data on graft interstitial fibrosis using biopsies at time of KTX and 1 year after. To our knowledge, all existing studies to date have been based on biopsies at time of KTX only.

Given our results and the continually increasing age of LD, we suggest generalizing pre-donation ABPM as part of LD selection. This approach would enable screening of LD with pre-existing HT, as well as those with BP at the upper limit, with white coat HT, masked HT or absence of dipping without HT. For all these patients, post-donation monitoring of BP is especially important, and allows for the reinforcement of lifestyle modifications (reduction of salt consumption to <5 g/day, increasing exercise, avoidance of alcohol and NSAIDs), and for the early introduction of antihypertensive treatment if necessary. In our center, we systematically perform ABPM at 6 months or 1-year post-donation in LD with BP at the upper limit or with no pre-donation dipping.

Our study has several limitations. First and foremost, it is a retrospective study, and residual confounding bias may remain. We have no data on graft rejection episodes, post-transplant infection, surgical complications, immunosuppression through levels, which are all variables that may impact recipient eGFR. Our population was mostly composed of Caucasian women, so our results cannot necessarily be generalized to other populations. For instance, our results may have been different if our population was mostly male. Office BP data was based on only 1 recorded measurement, and as our study is retrospective, we do not know if this was the mean or the best of 3 measurements, or if only 1 measurement was recorded. Furthermore, we have ABPM data for just over half of LD (112 patients/212), and analyses based on ABPM data are therefore derived from this subgroup, which reduces their statistical power. However, effect size for ABPM and office BP data was similar, and we expect that with more patients, analyses based on ABPM would be significant, with similar results than those shown for office BP. Furthermore, office BP and ABPM were well correlated in the subgroup of patients who had both. Due to a lack of biopsy data for patients transplanted before the 2010s, we were unable to perform precise analyses of the BANFF score or of other markers of nephroangiosclerosis (such as arteriolar hyalinosis, glomerular sclerosis and tubular atrophy).

When pre-donation HT is diagnosed in LD, it is treated, and donation is delayed until satisfactory BP control is achieved. We can hypothesize a possible bias, in which good BP control explains why LD HT shows no impact on recipient eGFR. However, this hypothesis cannot be formally verified in our retrospective study, and it would be unethical to conduct an interventional study in which HT, once identified, is not treated.

In conclusion, our study found that LD HT was not associated with a decrease in recipient eGFR at 6 months, 1 year, 5 years or 10 years after KTX, or with greater interstitial fibrosis on biopsies at time of KTX or 1 year after, after adjustment for confounding factors. These findings are reassuring and support the current practice of accepting well-controlled hypertensive patients for donation, with no additional risk for the recipients’ short-, medium- and long-term renal prognosis. Prospective studies, including larger numbers of patients and longer-term follow-up, are needed to confirm these results.

## Data Availability

The data analyzed in this study is subject to the following licenses/restrictions: data from the electronic patients records, and the living donor swiss registry (SOL-DHR). Requests to access these datasets should be directed to sophie.huegli@hopitalvs.ch.

## Supplementary material

SUPPLEMENTARY DATA SHEET 1 Relationship between pre-donation daytime APBM SBP (mmHg) and pre-donation OBPM SBP (mmHg) in the subgroup of LD who had both (N=112) ABPM: ambulatory blood pressure monitoring, SBP: systolic blood pressure, OBPM: Office Blood Pressure Monitoring, LD: living donors.

SUPPLEMENTARY DATA SHEET 2 Relationship between pre-donation daytime APBM DBP (mmHg) and pre-donation OBPM DBP (mmHg) in the subgroup of LD who had both (N=112) ABPM: ambulatory blood pressure monitoring, DBP: diastolic blood pressure, OBPM: Office Blood Pressure Monitoring, LD: living donors.

SUPPLEMENTARY DATA SHEET 3 BP status of LD prior to donation, according to OPBM and ABPM (N=112) HT on OBPM defined >140/90mmHg. HT on ABPM defined >130/80mmHg.
