# Supplementary figures and images for: The impact of living donor hypertension on later function of kidney graft in kidney transplantation
Source: Front Med (Lausanne). 2025 Dec 3;12:1686473. doi: 10.3389/fmed.2025.1686473 (PMC12710234; doi:10.3389/fmed.2025.1686473)

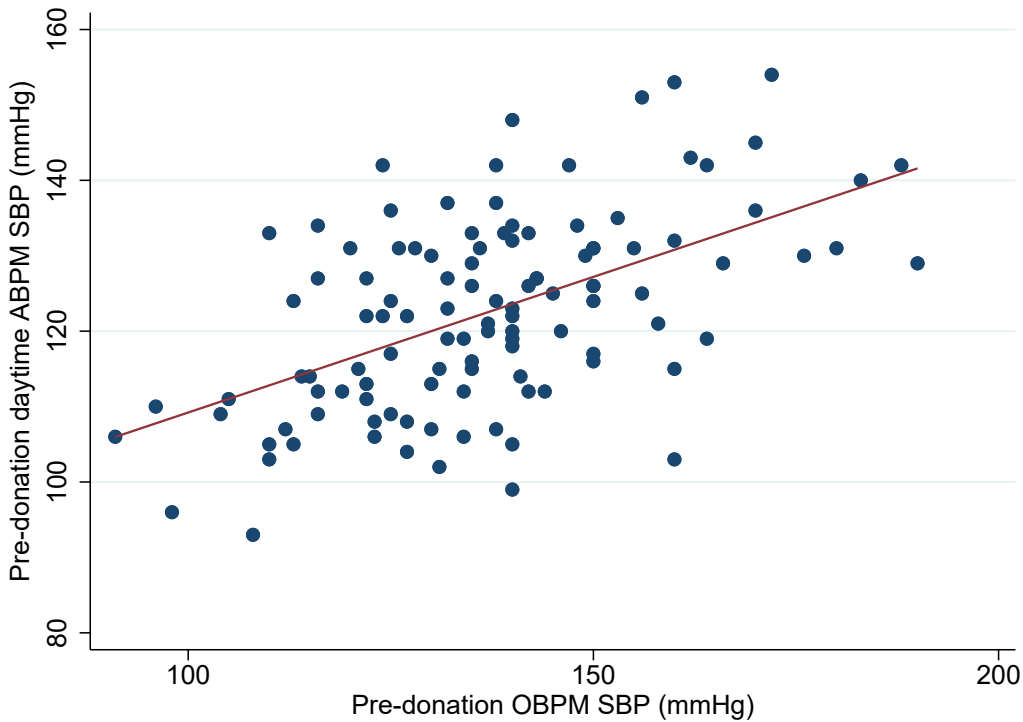

Supplement: SUPPLEMENTARY DATA SHEET 1 — Relationship between pre-donation daytime APBM SBP (mmHg) and pre-donation OBPM SBP (mmHg) in the subgroup of LD who had both (N=112) ABPM: ambulatory blood pressure monitoring, SBP: systolic blood pressure, OBPM: Office Blood Pressure Monitoring, LD: living donors. [file Data_Sheet_1.PDF]

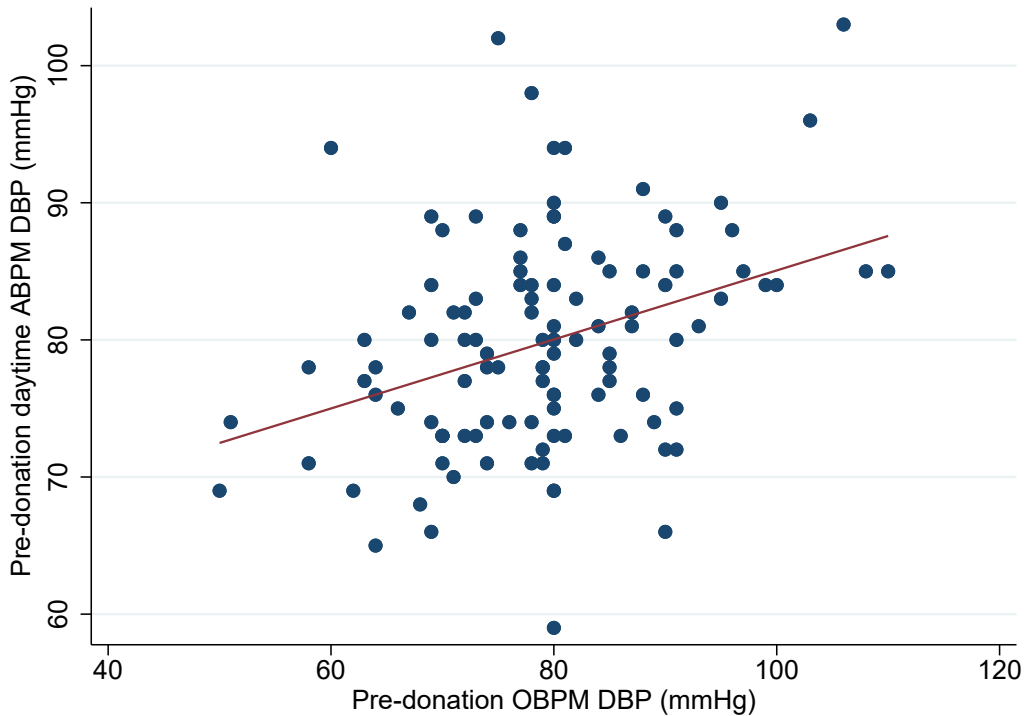

Supplement: SUPPLEMENTARY DATA SHEET 2 — Relationship between pre-donation daytime APBM DBP (mmHg) and pre-donation OBPM DBP (mmHg) in the subgroup of LD who had both (N=112) ABPM: ambulatory blood pressure monitoring, DBP: diastolic blood pressure, OBPM: Office Blood Pressure Monitoring, LD: living donors. [file Data_Sheet_2.PDF]
