# Supplementary material for: The impact of living donor hypertension on later function of kidney graft in kidney transplantation
Source: Front Med (Lausanne). 2025 Dec 3;12:1686473. doi: 10.3389/fmed.2025.1686473 (PMC12710234; doi:10.3389/fmed.2025.1686473)
Supplement: SUPPLEMENTARY DATA SHEET 3 — BP status of LD prior to donation, according to OPBM and ABPM (N=112) HT on OBPM defined >140/90mmHg. HT on ABPM defined >130/80mmHg. [file Data_Sheet_3.pdf]

| LD without HT on ABPM | LD with HT on ABPM | Total | |
|---|---|---|---|
| LD without HT on OBMP | **Normotensive**<br>33 (29.5%) | **Masked hypertension**<br>28 (25%) | 61 (54.5%) |
| LD with HT on OPBM | **White coat hypertension**<br>15 (13.4%) | **Sustained hypertension**<br>36 (32.1%) | 51 (45.5%) |
| Total | 48 (42.9%) | 64 (57.1%) | 112 (100%) |
